# Wilms tumor with inferior vena cava duplication: a rare case report

**DOI:** 10.1186/s12894-018-0401-0

**Published:** 2018-10-19

**Authors:** Feng Guo, Tianyou Li, Wei Liu, Gang Wang, Rui Ma, Rongde Wu

**Affiliations:** 1 Department of Pediatric Surgery, Shandong Provincial Hospital Affiliated to Shandong University, 324 Jingwu Road, Jinan, 250021 Shandong Province People’s Republic of China; 2 Shandong Medical Imaging Research Institute, Medical School of Shandong University, Jinan, Shandong Province People’s Republic of China

**Keywords:** Wilms tumor, Inferior vena cava duplication, Computed tomography

## Abstract

**Background:**

Wilms tumor is the most common renal tumor of childhood. Duplication of the inferior vena cava is an uncommon anomaly. In the present study, we present a case of Wilms tumor with the inferior vena cava duplication, which has not been reported previously.

**Case presentation:**

A 14-month-old female presented with an enlarging abdominal mass. Computed tomography imaging demonstrated a large mass in the right kidney, duplication of the inferior vena cava below the renal veins and compression of the right inferior vena cava caused by the enormous mass. A right radical nephrectomy was performed. Final pathology was consistent with Wilms tumor. Postoperative adjuvant chemotherapy was executed. Computed tomography imaging at 3 months postoperatively showed the right inferior vena cava played a dominant role and the left inferior vena cava was not detected clearly. During the follow-up of 18 months, no local recurrence or metastasis has been observed.

**Conclusion:**

It is important to recognize the case of Wilms tumor with the inferior vena cava duplication to avoid injury of retroperitoneal venous anomalies and life-threatening hemorrhage during surgery through preoperative computed tomography.

**Electronic supplementary material:**

The online version of this article (10.1186/s12894-018-0401-0) contains supplementary material, which is available to authorized users.

## Background

Wilms tumor (WT) is the most common renal tumor in childhood. Duplication of the inferior vena cava (IVC) is an uncommon anomaly. Although it is asymptomatic and often detected incidentally by imaging, IVC duplication may represent a hazard for bleeding during surgery, such as nephrectomy. In the present study, we present a case of WT with IVC duplication, which has not been reported previously.

## Case presentation

**Fig. 1:**
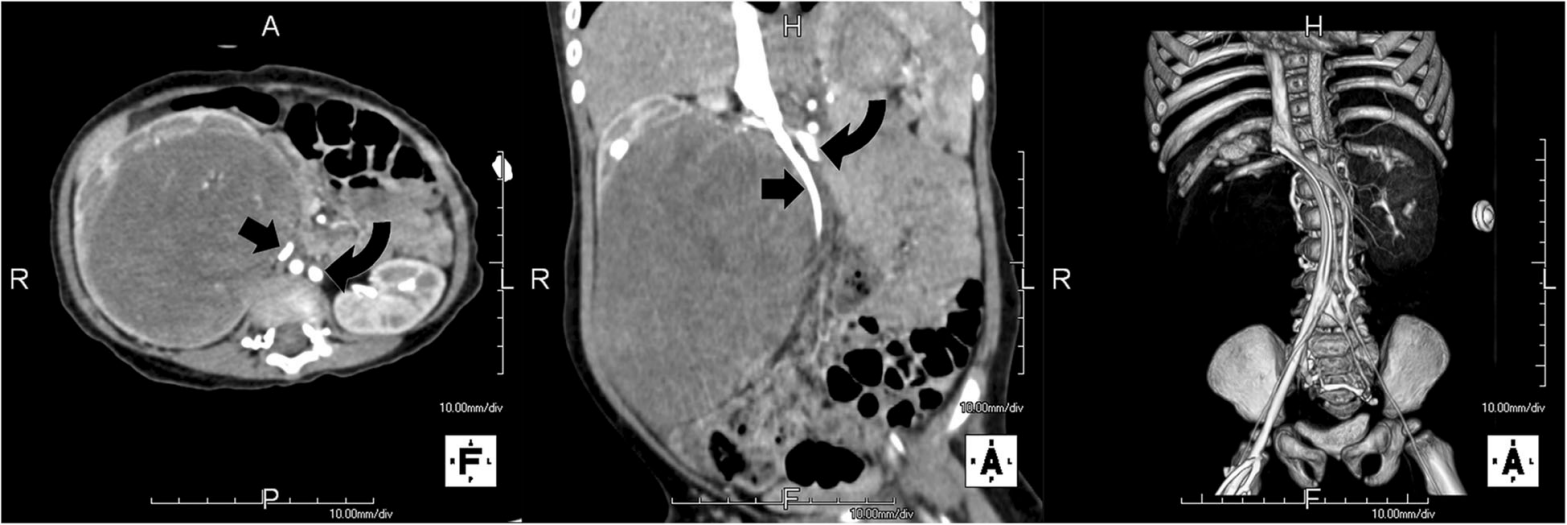
Axial, coronal and three-dimensional reconstruction computed tomography scan with contrast showing a large mass in the right kidney, duplication of the inferior vena cava below the renal veins and compression and displacement of the right inferior vena cava. Straight arrow denotes the right inferior vena cava, and curved arrow the left inferior vena cava

A 14-month-old female was referred to our hospital with a history of an enlarging abdominal mass noted by her parents for 3 days. Physical examination revealed an abdominal mass with clear and smooth margins extending from the right upper quadrant to the right hemipelvis. Routine blood tests were normal apart from mild anaemia and urine analysis did not show hematuria. Ultrasonography of the abdomen revealed a unilateral 10.8 × 7.2 × 9.2 cm solid tumor in the right kidney, whereas the contralateral kidney was normal. Computed tomography (CT) revealed a large lesion arising from the inferior aspect of the right kidney, occupying the right flank and extending across the midline. Enhanced CT detected duplication of IVC below the renal veins and compression and displacement of the right IVC caused by the enormous tumor (Fig. 1). An additional movie file shows this in more detail [see Additional file 1].

Neither intravascular extension nor invasion to adjacent organs and regional lymph nodes was detected by CT. Chest radiography was reported normal.

**Fig. 2:**
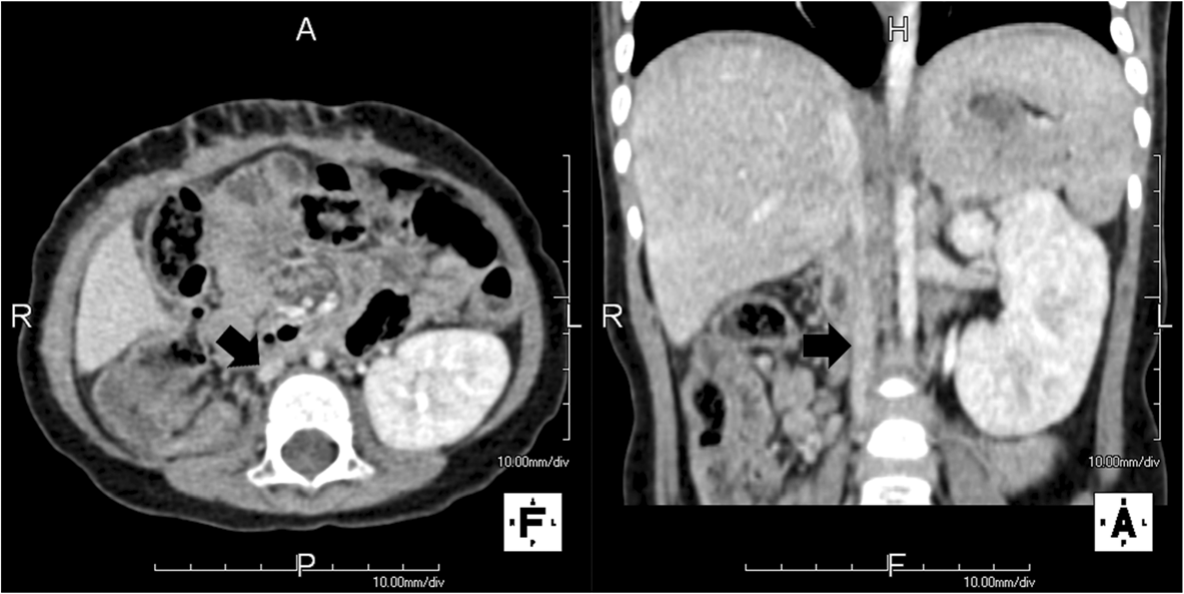
Axial and coronal computed tomography scan with contrast 3 months after operation showing the right inferior vena cava played a dominant role and the left inferior vena cava was not detected clearly. Straight arrow denotes the right inferior vena cava

With the presumptive diagnosis of WT, a right-sided radical nephrectomy was performed. Final pathology was consistent with favorable histology, stromal-predominant (60%) WT. The renal vessel and IVC were tumor free. The renal hilar and para-aortic lymph nodes were also free from tumor and the final pathological stage was Stage I. According to the regimen of the National Wilms’ Tumor Study Group 5, the patient received postoperative chemotherapy with dactinomycin and vincristine. CT imaging at 3 months postoperatively showed no evidence of residual or recurrent disease. Interestingly, the right IVC played a dominant role and the left IVC seemed to disappear in postoperative enhanced CT (Fig. 2). An additional movie file shows this in more detail [see Additional file 2]. During the follow-up of 18 months, no local recurrence or metastasis has been observed.

## Discussion

WT is the most common tumor of the urinary tract under the age of 15 [1]. Up to now, WT with IVC duplication has not been reported. The development of the IVC is a complex embryological process between weeks 6 and 10 of gestation, including the development, regression, anastomoses, and replacement of embryonic veins [2]. Double IVC is a rare anomaly, and occurs in 1.5% (range 0.2–3%) [3–5]. According to the caliber of the duplicated IVC and the preaortic trunk, IVC duplication has been classified into three types [4]. The classification may not be appropriate for the present case considering the notable changes of the caliber of the duplicated IVC before and after the operation. The compression of the right IVC caused by tumor resulted in the dominant venous drainage of the left IVC. After the tumornephrectomy, the right IVC gradually took over the venous drainage. Then the left IVC might start to regress and could not be detected at 3 months postoperative CT image. To our knowledge, this is the first detection of the postnatal regression of left IVC, which indicates that vena caval development might proceed not limited to the embryonic period.

Although IVC duplication is usually asymptomatic, it might have significant clinical implications. As an uncommon anomaly, the duplication of IVC can be misdiagnosed as a pathological lesion such as ureteric dilatation or lymphadenopathy on CT images. The left side of a double IVC might be interpreted erroneously as enlarged retroperitoneal lymph nodes [2, 6–8], which might induce preoperative overstaging by radiographic imaging in WT. Serious overstaging due to erroneous interpretation of the CT appearance of a double IVC has been reported in testicular tumor [6, 7].

A radical nephrectomy with lymph node sampling is the “gold standard” surgical protocol for unilateral WT [9–11]. However, retroperitoneal surgery such as nephrectomy might injure the anomalous venous structures that are in fact typically thin walled, dilated and tortuous [12]. Thus, it is vital to recognize the abnormal vasculature such as IVC duplication and avoid a life-threatening hemorrhage in retroperitoneal surgery or intervention [4, 13–16].

In addition to an en bloc resection of the tumor, lymph node sampling is another significant goal in WT surgery [17]. However, patients with anomalous venous anatomy might have unusual patterns of lymph node metastases, for the reason that the lymphatic drainage generally follows the vascular architecture [18]. Thus, lymph node sampling or dissection in a patient with a venous anomaly might be adjusted accordingly [7].

WT involves IVC invasion in 4–8% of cases [19], and caval extension of WT has been an important surgical challenge. Although tumor thrombus of renal vein or IVC was not found in the present case, extension of renal cell carcinoma into duplications of IVC and the retroaortic renal veins has been reported [18, 20–22].

CT is the most reliable technique for detecting tumor and retroperitoneal venous anomalies. Three-dimensional CT angiography can be used for detecting the duplication of IVC before surgery or other interventional procedures of the retroperitoneum are undertaken [23]. In WT, preoperative CT is also an important diagnostic tool and adjunct in assessing lymph node involvement to provide accurate treatment recommendations [24].

In our case, a plain chest X-ray was performed for the evaluation of pulmonary metastases and the report was normal. Considering the disadvantage of radiation exposure, a routine pulmonary CT that was still controversial [25] was not performed.

## Conclusion

WT with IVC duplication is extremely rare. Preoperative CT can detect potential venous anomalies in retroperitoneum. Appropriate care can be taken to avoid injury of retroperitoneal venous anomalies and life-threatening hemorrhage during surgery.

## Additional files

Additional file 1: Axial computed tomography sequential images with contrast before operation. (AVI 6664 kb)

Additional file 2: Axial computed tomography sequential images with contrast 3 months after operation. (AVI 5284 kb)

## Abbreviations

CT: Computed tomography
IVC: Inferior vena cava
WT: Wilms tumor
